# Prevalence of nosocomial infections in intensive care units and the role of the infection prevention and control team in implementing protective measures – a case study of a university hospital in Morocco

**DOI:** 10.3205/dgkh000644

**Published:** 2026-03-20

**Authors:** Mounir Arai, Mohamed Ouhadous, Sabah Salih, Halima Lajane, Rachid Gouifrane, Omar Abidi, Khalid Khaleq

**Affiliations:** 1 Laboratory of Care, Health and Sustainable Development (2S2D)-Team: Care and Biology-Health, Higher Institute of Nursing and Health Technical Professions (ISPITS), Casablanca, Morocco; 2 Laboratory of Chemistry-Biochemistry, Environment, Nutrition, and Health – Hassan II University, Faculty of Medicine and Pharmacy of Casablanca, Morocco

**Keywords:** prevalence, nosocomial infections, intensive care units, infection control, quality improvement, Morocco

## Abstract

**Introduction:**

Despite their small number of beds, intensive care units are major reservoirs of multidrug-resistant bacteria (MDRO) and experience a high frequency of nosocomial infections. This study aims to estimate their prevalence, identify risk factors, determine the microorganisms responsible, and highlight the preventive measures taken to control these risks.

**Methods:**

A cross-sectional questionnaire-based survey of the prevalence of nosocomial infections in all intensive care units in three university hospital centers, including all patients hospitalized for at least 24 hours.

**Results:**

Among the 50 patients included, 30% had a nosocomial infection, the most common of which was pneumonia. Of the 18 bacteria species isolated in 15 patients, 66.7% were MDRO. The most frequently isolated species was *Acinetobacter baumannii* (44.4%), with a resistance rate of 100% against imepenem.

**Conclusion:**

The survey identified the frequency, risk factors and microorganisms responsible for nosocomial infections. This approach helped to guide preventive measures and prioritize efforts, making it a pragmatic and appropriate solution for enhancing the efforts against these infections in contexts where resources are limited.

## Zusammenfassung

**Einleitung:**

Intensivstationen sind trotz ihres geringen Anteils an Betten ein großes Reservoir für multiresistente Bakterien (MDRO) und weisen eine hohe Häufigkeit nosokomialer Infektionen auf. Diese Studie zielt darauf ab, deren Prävalenz zu schätzen, Risikofaktoren zu identifizieren, die verantwortlichen Mikroorganismen zu bestimmen und Präventionsmaßnahmen zur Eindämmung der Risiken aufzuzeigen.

**Methoden:**

Es wurde eine Fragebogen-basierte Querschnittsuntersuchung zur Prävalenz nosokomialer bei allen Patienten, die mindestens 24 Stunden lang hospitalisiert waren, durchgeführt.

**Ergebnisse:**

Von 50 eingeschlossenen Patienten wiesen 30,0% eine nosokomiale Infektion auf. Die häufigste Lokalisation war die Lunge. Von 18 Bakterienspecies, die bei 15 Patienten isoliert wurden, waren 66,7% multiresistent. Die am häufigsten isolierte Spezies war *Acinetobacter baumannii* (44,4%) mit einer Resistenzrate von 100% gegen Imepenem.

**Schlussfolgerung:**

Die Untersuchung ermöglichte es, die Häufigkeit, die Risikofaktoren und die verantwortlichen Mikroorganismen zu identifizieren. Dieser Ansatz trug dazu bei, Präventionsmaßnahmen zu steuern die Kontrollmaßnahmen gegen diese Infektionen im Kontext von begrenzten Ressourcen zu verstärken.

## Introduction

Healthcare-associated infections (HAIs) are recognized worldwide as a public health problem, and are responsible for a significant increase in mortality, morbidity, and patient care costs [1], [2]. Their frequency and severity are particularly high in intensive care units (ICUs) due to the pathologies presented by patients, associated comorbidities, and the density of invasive interventions [3], [4]. Although ICUs generally account for only a small proportion of hospital beds, they are considered to be a significant reservoir of multidrug-resistant organisms (MDRO) and a place where healthcare-associated infections (HAIs) are very common [5], [6]. The prevalence is four to five times higher in intensive care and resuscitation units compared to other healthcare sectors, and is estimated at 22.4% in a French study [7], [8], [9], [10].

Among preventive measures, prevalence studies are a simple, quick, and inexpensive way to rapidly assess the extent of the problem, particularly in developing countries where resources are often limited [11], [12]. Regular prevalence surveys are a valuable tool for monitoring changes in infection rates over time, enabling comparisons between different units within the institution [13], [14]. When conducted at the hospital level, this approach also helps to raise awareness among healthcare workers (HCWs) about the challenges of HAIs and to increase the visibility of the infection control team, particularly when launching a prevention and control program. This approach also facilitates an initial assessment of the problems encountered in each unit, taking into account all types of infections and the diversity of patients. It thus makes it possible to target interventions more effectively and define priorities for treatment [15], [16].

The aim of this study is to estimate the prevalence of hospital-acquired infections (HAIs) in ICUs, identify the associated risk factors, determine the microorganisms responsible according to anatomical site, and highlight the efforts and actions taken by the infection prevention and control team in managing infection risk in ICUs.

## Materials and methods

### Study design and setting 

A cross-sectional survey of the prevalence of HAIs in all ICUs of three hospitals belonging to a university hospital center in Morocco was conducted in 2024.

### Study population 

The study included all patients hospitalized for at least 24 hours in one of the ICUs and who were present on the day of the survey. Newborns less than two days old were excluded.

### Data collection 

The criteria for defining HAI were based on the WHO definitions [17]. The data were collected using a questionnaire by doctors and nurses who had received training prior to the day of the survey.

The data were collected from medical records with the support of the attending doctor and nurse. The diagnosis of HAI was made by the investigator and confirmed and documented in collaboration with the doctors in the department.

Statistical analysis was performed using SPSS v. 16 with a significance threshold set at 5%. It consisted of descriptive, bivariate, and multivariate analyses.

### Variables 

The questionnaire consisted of two sections. The first section included general information about the patients (age, gender, length of stay, immunosuppression, ASA score, presence of an invasive medical device, surgical procedure, etc.).The second section was intended only for patients who had an HAI (site of infection, isolated pathogens, etc.).

### Ethical considerations 

Anonymity and confidentiality were respected. Patients were identified using a hospitalization number in order to collect the results of bacteriological diagnostics. The study was approved by the institution's ethics committee, file no. 04/2025.

## Results

### Characteristics of the study population 

The survey involved 50 patients who were hospitalized on the day of the survey for at least 24 hours in intensive care. The study population consisted of 19 (41.3%) female patients and 27 (58.7%) male patients (44.7%). The 45- to 59-year age group represented the largest proportion at over 26%. Interdepartmental transfers from other hospital wards accounted for 44.0% of ICU admissions, whereas direct admissions were less frequent. Among the patients hospitalized on the day of the survey, 71.4% did not have underlying immunodeficiency. More than half of the patients for whom the ASA score was specified had a score ≥3; no patients had a score of 5. Among the 50 patients whose date of hospitalisation was specified, 21 (75.6%) were hospitalized for <3 days, 14 (28.0%) had been hospitalized for ≥12 days. The average hospital stay was 10.7 days (±12.2). Diagnoses upon admission were medical (64%), emergency surgery 12%, other 12%.

### Invasive devices 

All patients had at least one invasive device, and 92% had two or more devices. Half of the patients (50%) were mechanically ventilated (further details in Table 1 (Tab. 1)).

### Anti-infectives 

78% were prescribed anti-infective treatment (Table 2 (Tab. 2)). 

### Nosocomial infections 

15 patients (30.0%) developed a nosocomial infection on different anatomical site (Table 3 (Tab. 3)). 

### Bacterial etiology of nosocomial infections 

*Acinetobacter (A.) baumannii* was the most common pathogen, and all isolates were resistant to imipenem (Table 4 (Tab. 4)). 

## Discussion

The prevalence of HAIs in our ICUs (30%) is consistent with findings of studies conducted in sever al countries, where reported prevalence ranged from 3.5% in Iran [18], 48.7% in Turkey [19], 13% in Serbia [20][20], 43% in Australia. Higher prevalence rates of up to 60% have been reported in Asia and the Middle East [21]. Similarly, the EPIC (European Prevalence of Infection in Intensive Care) study reported a prevalence of 44.8% [22]. The differences can be explained by variations in the study periods, as well as by improvements in working conditions and healthcare practices [22], [23]. In addition, the impact of HAI control and surveillance programs plays a key role. According to the latest World Health Organization (WHO) global report on infection prevention and control (2022), well-orchestrated infection control programs can reduce the incidence of nosocomial infections up to 70% [24].

On the day of the survey, all patients in ICUs had at least one invasive device, which are the main cause of HAIs, particularly when handling and hygiene rules are not strictly followed [25], [26]. In addition, HAIs associated with invasive devices affect between 24.3% and 27.6% of patients in ICUs, demonstrating their significant impact on the risk of infection in intensive care [27].

Ventilator-associated pneumonia (VAP) was the most common (18%), followed by catheter-related infections and bacteraemia. The most frequent sites of infection were consistent with those reported in the literature [28], [29]. VAP is the major factor in the emergence of nosocomial pneumonia, and all studies on this subject show that the number of these infections increases with the duration of ventilation [30]. A retrospective study (567 patients) showed that the risk of developing nosocomial pneumonia increases by 1% for each additional day of ventilation [31]. Several studies have also shown that the risk of developing pneumonia acquired under mechanical ventilation is highest between the 8th and 10th day of ventilation [32].

*A. baumannii*, the main bacterium isolated in this study, is known as an opportunistic pathogen frequently involved in outbreaks in ICUs and are often associated with contamination of ventilation and respiratory support equipment [33], [34]. In a large international study, Acinetobacter species were responsible for an average of 8.8% of Gram-negative infections in ICUs on all continents, and for more than 19% in Asian ICUs [35].

On the day of the survey, 78% of patients received at least one anti-infective agent. Penems were the most commonly prescribed anti-infective agents (48.7%). The increased prescription of carbapenem in recent years may have contributed to the rising incidence of extended-spectrum ß-lactamase (ESBL)-producing Enterobacteriaceae and high-level cephalosporinase-producing strains. [36], [37], [38].

Following this investigation, a prevention program was implemented in all ICUs at the university hospital. Recent studies [39], [40] have shown that an infection control program based on practical and targeted interventions can significantly improve the quality of care and reduce HAIs. This system is based on three main areas: monitoring HAIs (surveillance), promoting standard precautions, and strengthening healthcare workers' skills in hospital hygiene, accompanied by recommendations for controlling the risk of infection in intensive care units. Surveillance makes it possible to quickly identify any increase or change in infections while also assessing the effectiveness of preventive measures [41], [42]. In the present study, surveillance was implemented by two hygienists in close collaboration with the institution's hygiene team, according to a validated and approved internal procedure.

The second action was the development of a guide to preventing infection risk in intensive care. The idea arose after evaluating the results of the survey. HCWs are confronted daily with repetitive, increasingly complex, and varied care situations in units considered to be a significant reservoir of MDRO in a place where HAIs are very common. The guide takes the form of technical data sheets on hospital hygiene, and the main topics covered include standard and additional precautions, disinfecting cleaning equipment, managing an epidemic, and controlling the spread of MDRO. The main beneficiaries were medical and nursing staff, as well as students. 

The third step was simultaneously conducted to improve the use of antibiotics by organizing a masterclass in antibiotic therapy for healthcare staff. The aim was to rationalize the use of antibiotics in intensive care, given that controlling the spread of MDRO and the proper use of antibiotics are the subject of numerous current recommendations.

### Limitations

A cross-sectional study only provides a snapshot of the situation. Nevertheless, these surveys are cost-effective and allow a large amount of information to be collected in a short period of time compared to incidence studies. Furthermore, inter-observer variability in the interpretation of infection criteria may affect the accuracy of the results. To limit this bias, standardized training was provided to assessors to harmonize the application of diagnostic criteria, and a precise protocol was put in place to ensure consistency of observations during each visit.

## Conclusions

This prevalence survey contributes to efforts combating HAIs in intensive care, and can help improve the quality of care, reduce the prevalence of nosocomial infections, and ensure safer and more effective care for the patients. It also identifies areas for improvement in order to optimise infection prevention and management in ICUs.

## Notes

### Competing interests

The authors declare that they have no competing interests.

### Ethical approval

The study was approved by the institution's ethics committee, file no. 04/2025.

### Funding

None.

### Authors’ ORCIDs

- Arai M: https://orcid.org/0000-0002-4936-2403
- Ouhadous M: https://orcid.org/0000-0001-9790-7140
- Lajane H: https://orcid.org/0000-0001-6320-4949
- Gouifrane R: https://orcid.org/0000-0003-0810-6275
- Salih S: https://orcid.org/0009-0004-5721-8120
- Abidi O: https://orcid.org/0000-0002-1105-4798
- Khaleq K : https://orcid.org/0009-0004-0755-162X

## Figures and Tables

**Table 1:**
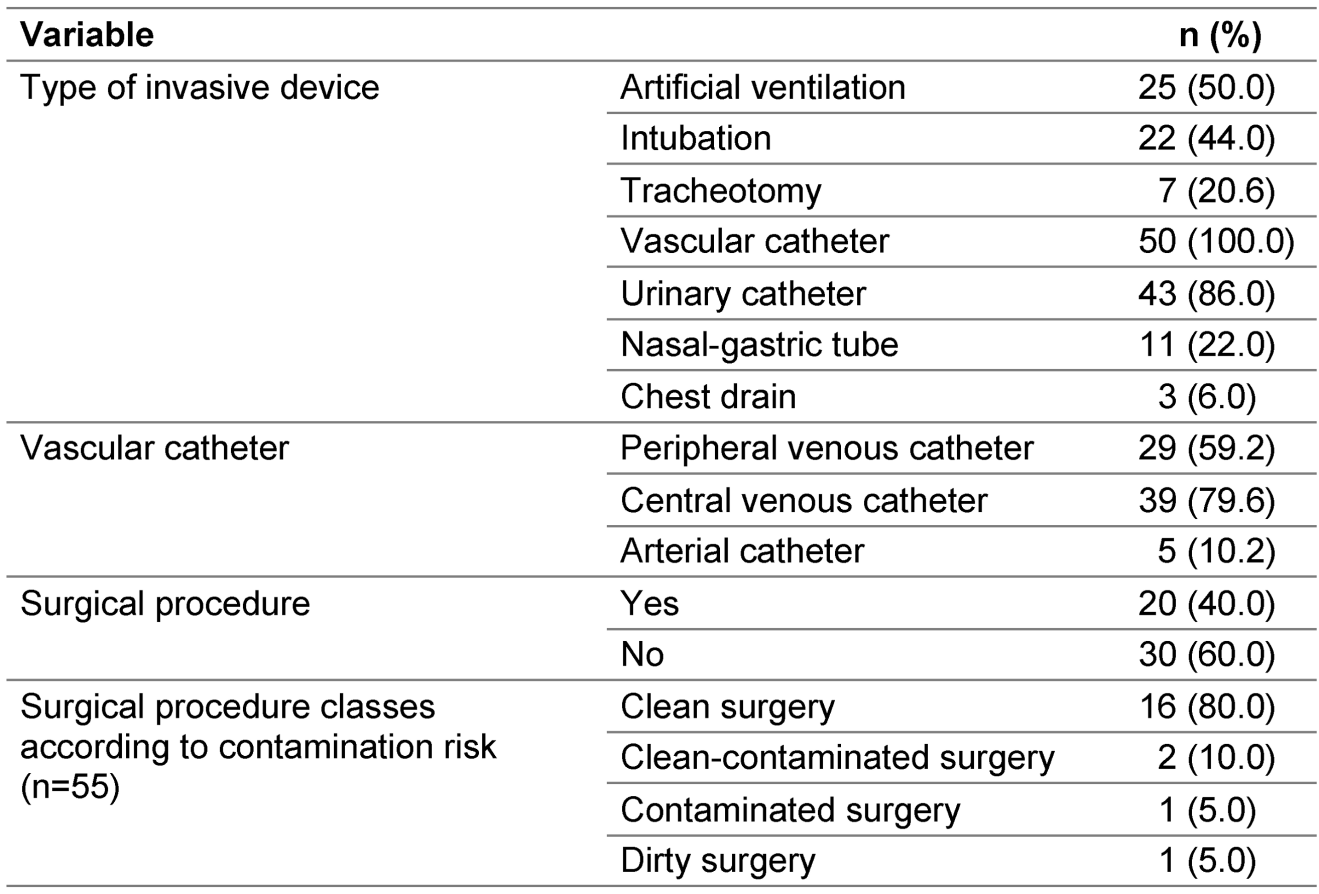
Invasive devices and surgical procedures

**Table 2:**
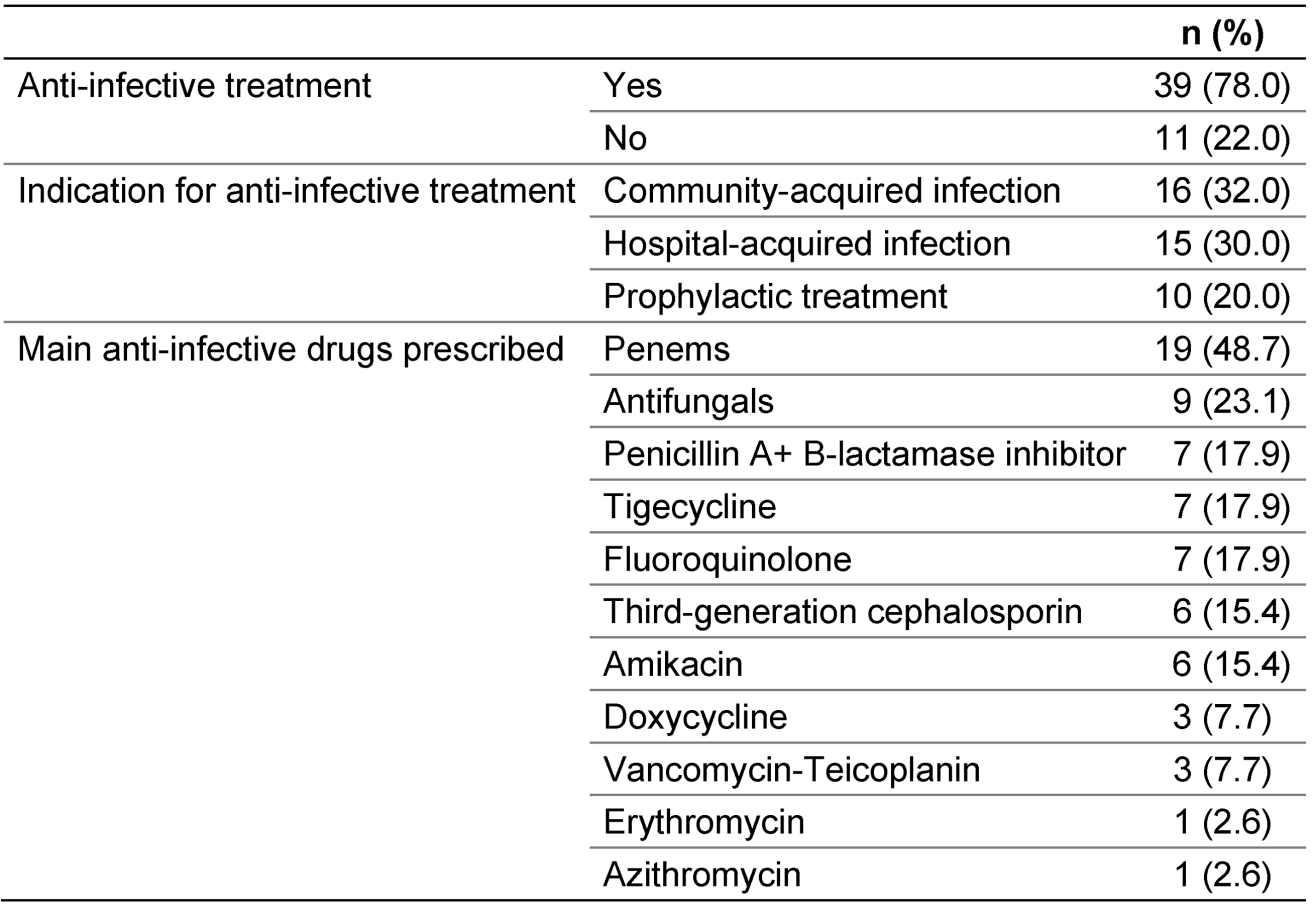
Reasons for anti-infective treatment (n=50)

**Table 3:**
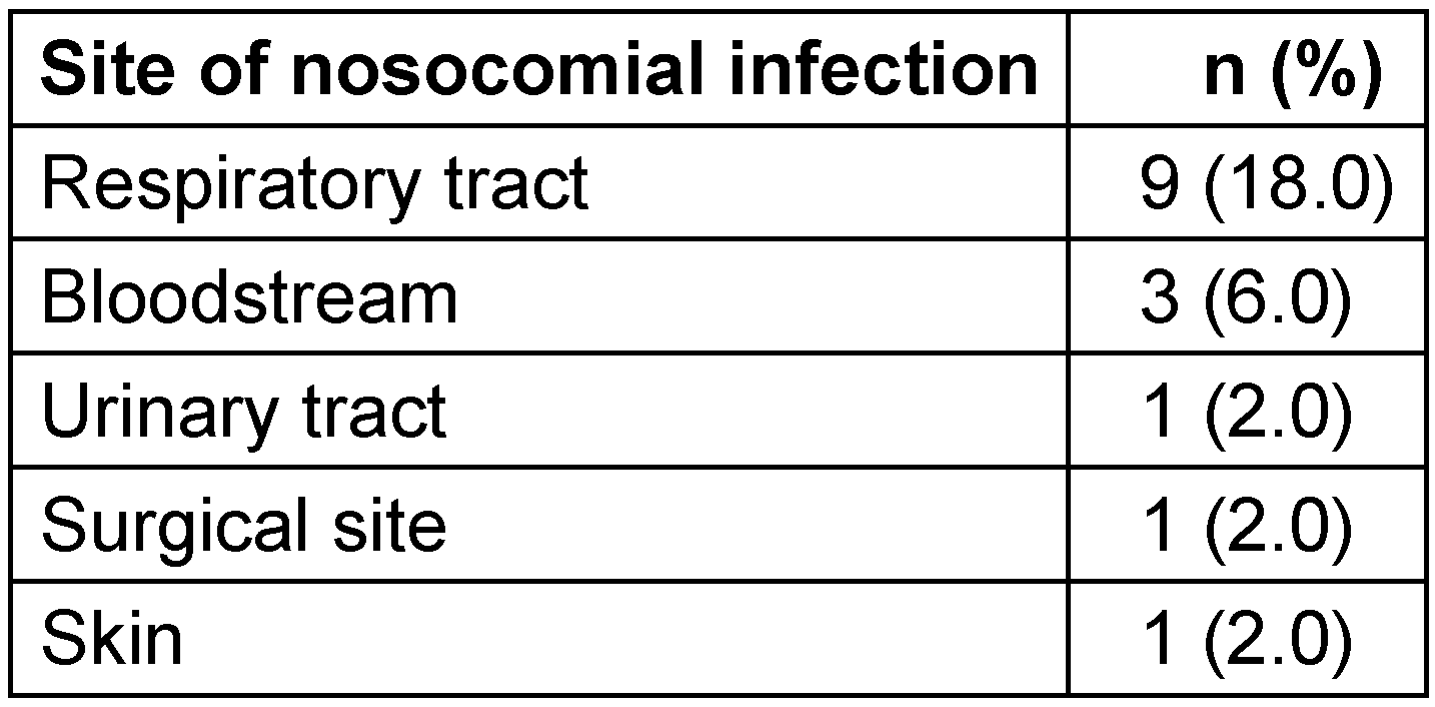
Incidence of nosocomial infection sites in the overall cohort (n=50)

**Table 4:**
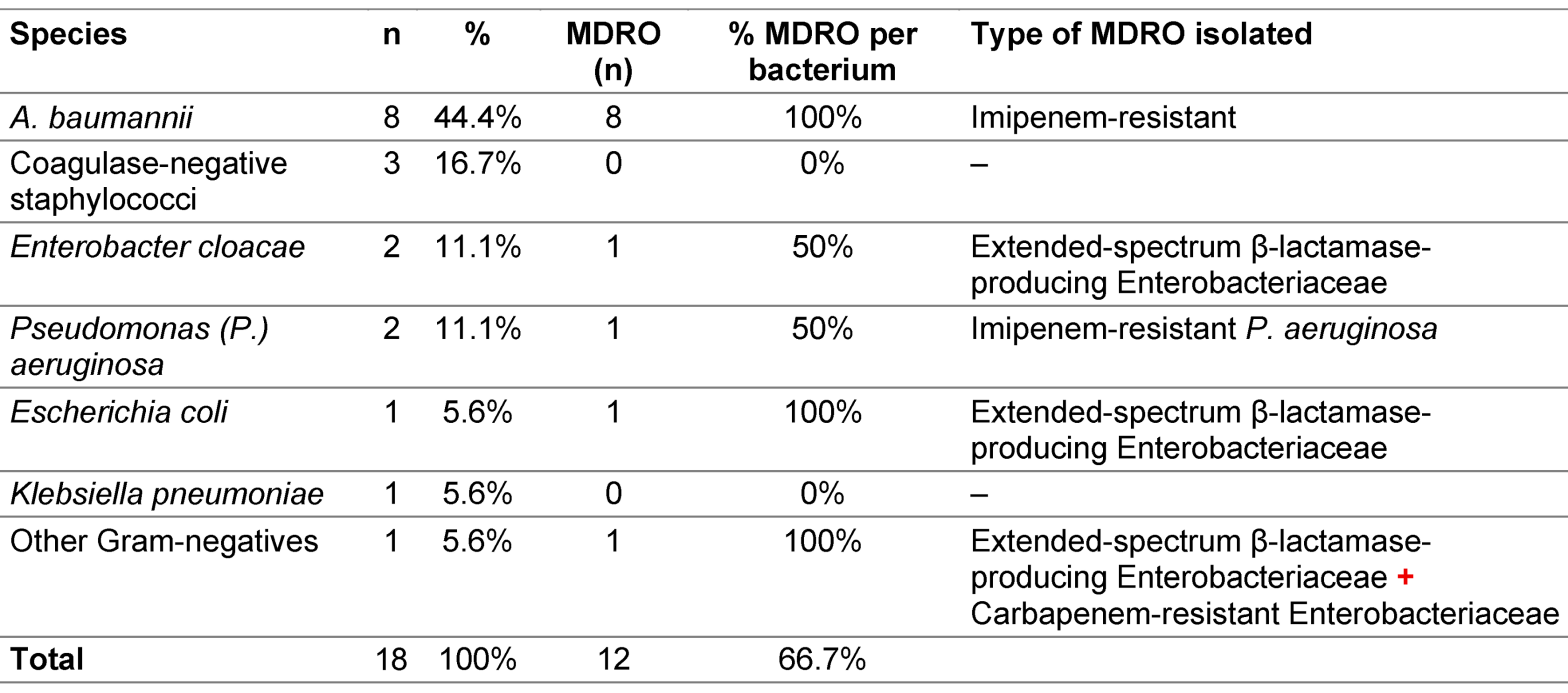
Distribution of the main bacteria responsible for nosocomial infections
